# Ultrasound-Assisted Extraction and Identification of Natural Antioxidants from the Fruit of *Melastoma sanguineum* Sims

**DOI:** 10.3390/molecules22020306

**Published:** 2017-02-18

**Authors:** Tong Zhou, Dong-Ping Xu, Sheng-Jun Lin, Ya Li, Jie Zheng, Yue Zhou, Jiao-Jiao Zhang, Hua-Bin Li

**Affiliations:** 1 Guangdong Provincial Key Laboratory of Food, Nutrition and Health, School of Public Health, Sun Yat-Sen University, Guangzhou 510080, China; zhout43@mail2.sysu.edu.cn (T.Z.); xudp@mail2.sysu.edu.cn (D.-P.X.); liya28@mail2.sysu.edu.cn (Y.L.); zhengj37@mail2.sysu.edu.cn (J.Z.); zhouyue3@mail2.sysu.edu.cn (Y.Z.); zhangjj46@mail2.sysu.edu.cn (J.-J.Z.); 2 Zhongshan Center for Disease Control and Prevention, Zhongshan 528403, China; zscdclsj@163.com; 3 South China Sea Bioresource Exploitation and Utilization Collaborative Innovation Center, Sun Yat-Sen University, Guangzhou 510006, China

**Keywords:** *Melastoma sanguineum* Sims, fruit, antioxidant, ultrasound-assisted extraction, response surface methodology, phenolic compounds

## Abstract

The fruit of *Melastoma sanguineum* Sims is an edible and sweet wild fruit. In our previous study, the fruit was found to have a strong antioxidant property. In this study, an ultrasound-assisted extraction (UAE) method was developed to extract natural antioxidants from the fruit of *Melastoma sanguineum* Sims, and a response surface methodology was used to optimize the conditions of UAE to maximize the extraction efficiency. The influence of five independent extraction parameters (ethanol concentration, solvent/material ratio, extracting time, temperature, and ultrasound power) on the extraction efficiency were investigated using a single factor experiment, and then a central composite rotatable design was used to investigate the interaction of three key parameters. The results showed that the optimal extraction conditions were 42.98% ethanol, 28.29 mL/g solvent/material ratio, 34.29 min extracting time, 60 °C temperature, and 600 W ultrasound power. Under these conditions, the Trolox equivalent antioxidant capacity (TEAC) value of the extracts was 1074.61 ± 32.56 μmol Trolox/g dry weight (DW). Compared with conventional maceration (723.27 ± 11.61 μmol Trolox/g DW) and Soxhlet extraction methods (518.37 ± 23.23 μmol Trolox/g DW), the UAE method improved the extraction efficiency, in a shorter period of time. In addition, epicatechin gallate, epicatechin, rutin, epigallocatechin, protocatechuic acid, chlorogenic acid, and quercetin, were identified and quantified in the fruit extracts of *Melastoma sanguineum* Sims by UPLC-MS/MS.

## 1. Introduction

Free radicals include reactive oxygen species (ROS), reactive sulfur species (RSS), and reactive nitrogen species (RNS) [1]. They are generated as a part of normal cellular metabolism. However, many external factors (smoking, drugs, pesticides, environmental pollutants, radiation, and industrial solvents) can increase the production of free radicals [2]. The overproduction of free radicals can result in oxidative stress, which is involved in many chronic and degenerative diseases, such as atherosclerosis, coronary heart diseases, diabetes mellitus, neurodegenerative diseases, ageing, and cancer [3,4,5]. Several epidemiological studies have verified that a high intake of vegetables and fruits are related with a reduced risk of many chronic diseases, such as hypertension, coronary heart disease, and cancer [6,7]. These health benefits might be attributable to the presence of antioxidants in vegetables and fruits. The antioxidants from plant sources are able to scavenge excess free radicals, inhibit lipid oxidation, and reduce the hydroperoxide formation, which could protect an organism against oxidative stress [8]. Some antioxidants from plant sources have been proved to have anti-aging actions, anticancer actions, and protective actions against cardiovascular diseases, obesity, diabetes mellitus, and neurodegenerative diseases [9]. Therefore, the antioxidants from plant sources can be used as potential agents for preventing and treating oxidative stress-related diseases [10]. In addition, natural antioxidants can be used as food additives for preventing the oxidative deterioration of food in the food industry [11]. However, nowadays, the application of natural antioxidants is not widely used in the food industry because of their low production and expensive price [12]. Therefore, in order to obtain more natural antioxidants, it is necessary to develop an effective way to extract antioxidants with a strong antioxidant activity from natural products.

*Melastoma sanguineum* Sims is a genus of shrub in the family Melastomataceae, and widely distributed in China, India, and Malaysia [13]. It is a often used as a garden pot plant due to its attractive purple red flowers. Its fruit are cup shaped and are 1.2–1.6 cm in length, and the top of fruit is wider than its base. The fruit is covered with red, long, hard, and coarse hair. The pulp is sweet and edible [14]. This fruit is usually consumed by people, and is used to help digestion in folk medicine. In recent years, the biological activities of *Melastoma sanguineum* Sims have been reported. Ik-Soo et al. found that the extract of aerial parts of *Melastoma sanguineum* Sims could inhibit the formation of advanced glycation end-products and reduce the activity of aldose reductase, thus illustrating potential for delaying and preventing diabetic complications [15]. In addition, *Melastoma sanguineum* Sims, together with *Embelia laeta* and *Paeonia ostii*, could be used to treat osteomyelitis and inflammation [16]. In our previous study, the fruit of *Melastoma sanguineum* Sims was found to have an extremely strong antioxidant property, which indicated that the fruit of *Melastoma sanguineum* Sims could be a rich source of natural antioxidants [17]. In order to fully utilize the fruit of *Melastoma sanguineum* Sims, developing an efficient method to extract its antioxidants and identify the components of these extracts, is necessary.

At present, the methods used to extract natural antioxidants from natural products include conventional (e.g., maceration, steam distillation, and Soxhlet extraction) and non-conventional (e.g., ultrasound-assisted extraction, microwave-assisted extraction, supercritical fluid extraction, and subcritical water extraction) extraction methods [18,19]. Compared with conventional extraction methods, non-conventional extraction methods are usually time-saving and require less organic solvent [20,21]. Therefore, non-conventional extraction methods are becoming more and more popular, especially ultrasound-assisted extraction (UAE). UAE is an effective and environmentally friendly extraction method. Ultrasound possesses a mechanical effect that can promote eddy and internal diffusion, thus increasing the mass transfer and penetration of the solvent into the sample matrix [22]. Cavitation induced by ultrasound can break cell walls, accelerating the release of the contents [23,24]. Furthermore, UAE is simple and inexpensive, and can be applied to industrial production on a large scale [25]. In this study, an UAE method was developed for extracting natural antioxidants from the fruit of *Melastoma sanguineum* Sims.

Many extraction parameters (e.g., concentration of solvent, solvent/material ratio, temperature, extracting time, and ultrasound power) can significantly affect the extraction efficiency of UAE, and the parameters could also interact with each other [26,27,28,29]. Therefore, it is important to optimize these parameters for obtaining high extraction efficiency. Response surface methodology (RSM) is a frequently-used tool for optimizing the extraction parameters [30]. RSM is an effective mathematical and statistical tool which is based on the fit of a polynomial equation to the experimental data, which then makes statistical previsions [31]. It can not only investigate the effect of independent parameters, but also the interaction of several parameters [32]. In the present study, single-factor experiments were adopted to investigate the effects of five independent extraction parameters (ethanol concentration, solvent/material ratio, extracting time, temperature, and ultrasound power) on the extraction efficiency of natural antioxidants from the fruit of *Melastoma sanguineum* Sims, and a RSM with a five-level, three-variable central composite rotatable design (CCRD) was then adopted, in order to study the interaction of three key extraction parameters. In addition, the extraction efficiencies of UAE and two conventional extraction methods (maceration and Soxhlet extraction) were compared. Furthermore, the phenolic compounds in the extract, obtained under the optimal extraction conditions, were identified and quantified by liquid chromatography-tandem mass spectrometry.

## 2. Results and Discussion

### 2.1. Single Factor Experiment Analysis

#### 2.1.1. Effect of Ethanol Concentration

**Figure 1:**
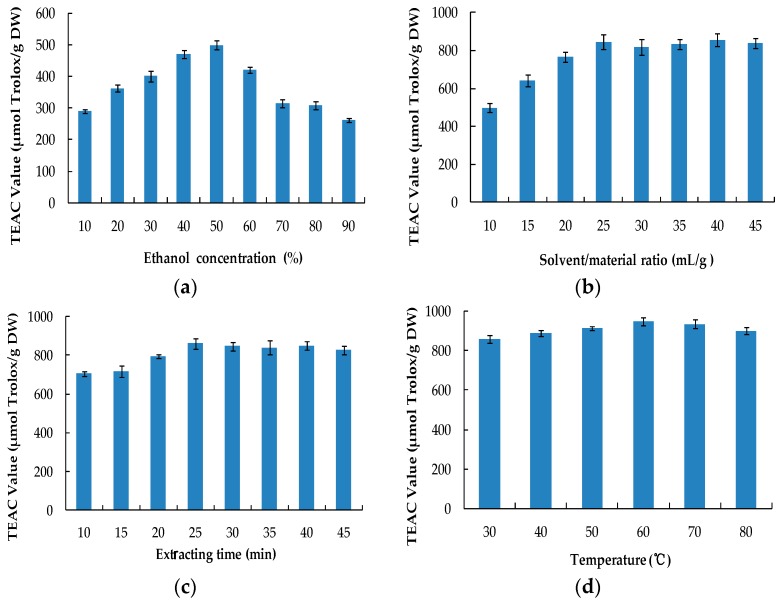

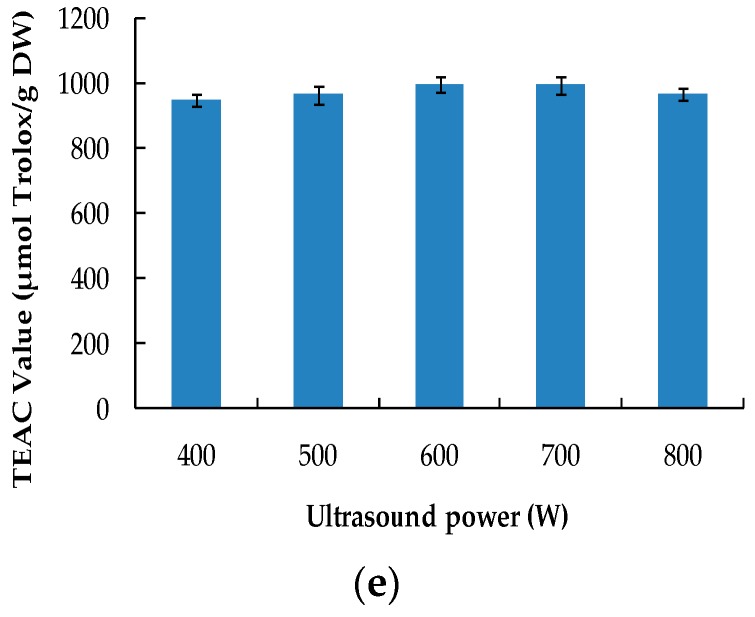
Effects of ethanol concentration (**a**); solvent/material ratio (**b**); extracting time (**c**); temperature (**d**) and ultrasound power (**e**) on the antioxidant activity of the extracts.

Due to the cheap and nontoxic characteristics of ethanol and water, aqueous ethanol is frequently applied to extract natural antioxidants from natural products [30,31]. In this study, the effect of ethanol concentration on extraction efficiency was studied under the following conditions: solvent/material ratio of 10 mL/g, extracting time of 30 min, temperature at 30 °C, and ultrasound power of 400 W. As shown in Figure 1a, when the ethanol concentration was increased from 10% to 50% (*v*/*v*), the antioxidant activity gradually increased (*p* < 0.05). When the ethanol concentration reached 50%, the antioxidant activity reached its peak. Following this, with the increase of ethanol concentration, the antioxidant activity gradually decreased (*p* < 0.05). According to the “like dissolves like” theory [33], 50% ethanol might have the most suitable polarity for antioxidant components in the fruit of *Melastoma sanguineum* Sims. Thus, in subsequent experiments, 50% ethanol was used as the extraction solvent.

#### 2.1.2. Effect of Solvent/Material Ratio

The impact of the solvent/material ratio on the extraction efficiency of natural antioxidants from the fruit of *Melastoma sanguineum* Sims was investigated with 50% ethanol, 30 min extraction time, 30 °C temperature, and 400 W ultrasound power. The results are showed in Figure 1b. When the solvent/material ratio increased from 10 mL/g to 25 mL/g, the antioxidant activity gradually increased (*p* < 0.05). When the solvent/material ratio reached 25 mL/g, the antioxidant activity reached its peak. When the solvent/material ratio increased from 25 mL/g to 45 mL/g, no significant change was observed. Generally, a higher solvent/material ratio results in a greater concentration difference, that can then promote mass transfer and accelerate the dissolution of solute. However, when the dissolution process reaches its equilibrium, further increasing the solvent/material ratio doesn’t improve the extraction efficiency [34,35]. Thus, 25 mL/g solvent/material ratio was applied in the subsequent experiments.

#### 2.1.3. Effect of Extracting Time

The effect of the extracting time on the extraction efficiency of natural antioxidants from the fruit of *Melastoma sanguineum* Sims was investigated with 50% ethanol, 25 mL/g solvent/material ratio, 30 °C temperature, and 400 W ultrasound power. The corresponding results are shown in Figure 1c. When the extracting time increased from 10 min to 25 min, the antioxidant activity increased (*p* < 0.05). However, no significant difference was observed from 25 min to 40 min. When the extracting time was prolonged to 45 min, the antioxidant activity slightly decreased (*p* > 0.05). The results show that ultrasound could accelerate the release of antioxidants from the material in a short period of time. However, as the ultrasound time is prolonged, ultrasound might destroy the antioxidants, resulting in the decrease of antioxidant activity [36]. Therefore, 25 min was chosen for the subsequent experiments.

#### 2.1.4. Effect of Extracting Temperature

The effect of the extracting temperature on the extraction efficiency of antioxidants from the fruit of *Melastoma sanguineum* Sims was studied with 50% ethanol, 25 mL/g solvent/material ratio, 25 min extracting time, and 400 W ultrasound power. As shown in Figure 1d, when the extracting temperature increased from 30 °C to 60 °C, the antioxidant activity increased (*p* < 0.05). As the temperature rose from 60 °C to 80 °C, the antioxidant activity significantly decreased (*p* < 0.05). The reason for this might be that a higher temperature initially promotes the diffusion, but when the temperature exceeds 60 °C, some heat labile antioxidant components are destroyed [37]. Therefore, 60 °C was the optimal extracting temperature.

#### 2.1.5. Effect of Ultrasound Power

The effect of ultrasound power on the extraction efficiency of antioxidants from the fruit of *Melastoma sanguineum* Sims was studied with 50% ethanol, 25 mL/g solvent/material ratio, 25 min extracting time, and 60 °C temperature. The corresponding results are shown in the Figure 1e. When the ultrasound power rose from 400 W to 600 W, the antioxidant activity increased significantly (*p* < 0.05). As the ultrasound power rose from 600 W to 800 W, the antioxidant activity slightly decreased (*p* > 0.05). The results indicate that the extraction efficiency was improved with the increasing ultrasound power, but when the ultrasound power exceeded 600 W, some antioxidant components were possibly degraded, which was consistent with the other report [38]. It should be pointed out that the ultrasound power was only the energy input to the generator, and that the actual ultrasonic energy introduced in the system was 12.5, 16.7, 19.6, 24.3, and 27.1 W/L, in response to the energy inputs of 400, 500, 600, 700, and 800 W, respectively.

### 2.2. Optimization by Response Surface Methodology

#### 2.2.1. Experimental Design and Results

According to the results of the single factor experiment, the effects of ethanol concentration, solvent/material ratio, and extracting time on the extraction efficiency, were more significant than those of the other factors. Therefore, they were further optimized by RSM under the conditions of a 60 °C extracting temperature and an ultrasound power of 600 W. A three-variable, five-level central composite rotatable design (CCRD) was adopted in RSM, and 50% ethanol, 25 mL/g solvent/material ratio, and 25 min extracting time, were selected as the middle level. The detailed experimental design, actual value, and predicted value, are shown in Table 1. The results show that the actual antioxidant activity changed from 591.72 to 1067.81 μmol Trolox/g DW.

#### 2.2.2. Fitting the Model

The results of Table 1 were analyzed using multiple regression fitting, and a quadratic polynomial regression model equation of the TEAC value (Y) to ethanol concentration (X_1_), solvent/material ratio (X_2_) and extracting time (X_3_) was obtained. The regression model equation is shown in Equation (1).

Y = 998.34 − 110.31X_1_ + 28.74X_2_ + 76.47X_3_ + 2.4X_1_X_2_ + 6.26X_1_X_3_ + 2.35X_2_X_3_ − 73.77X_1_^2^ − 44.35X_2_^2^ − 39.2X_3_^2^ (1)

The reliability of this quadratic polynomial model was analyzed by an analysis of variance (ANOVA), and the results of the ANOVA are shown in Table 2. The high *F* value (40.59) and low *p* value (*p* < 0.0001) of this model showed that the regression model was reliable. In addition, the low *F* value (1.32) and high *p* value (0.385) of “lack of fit”, indicated that the “lack of fit” was non-significant, which further confirmed the reliability of this regression model. The determination coefficient (R^2^) was 0.973, and the adjusted determination coefficient (Adj. R^2^) was 0.949, which indicated that the regression model could explain 94.9% of the response value variations.

#### 2.2.3. Response Surfaces Analysis

**Figure 2:**
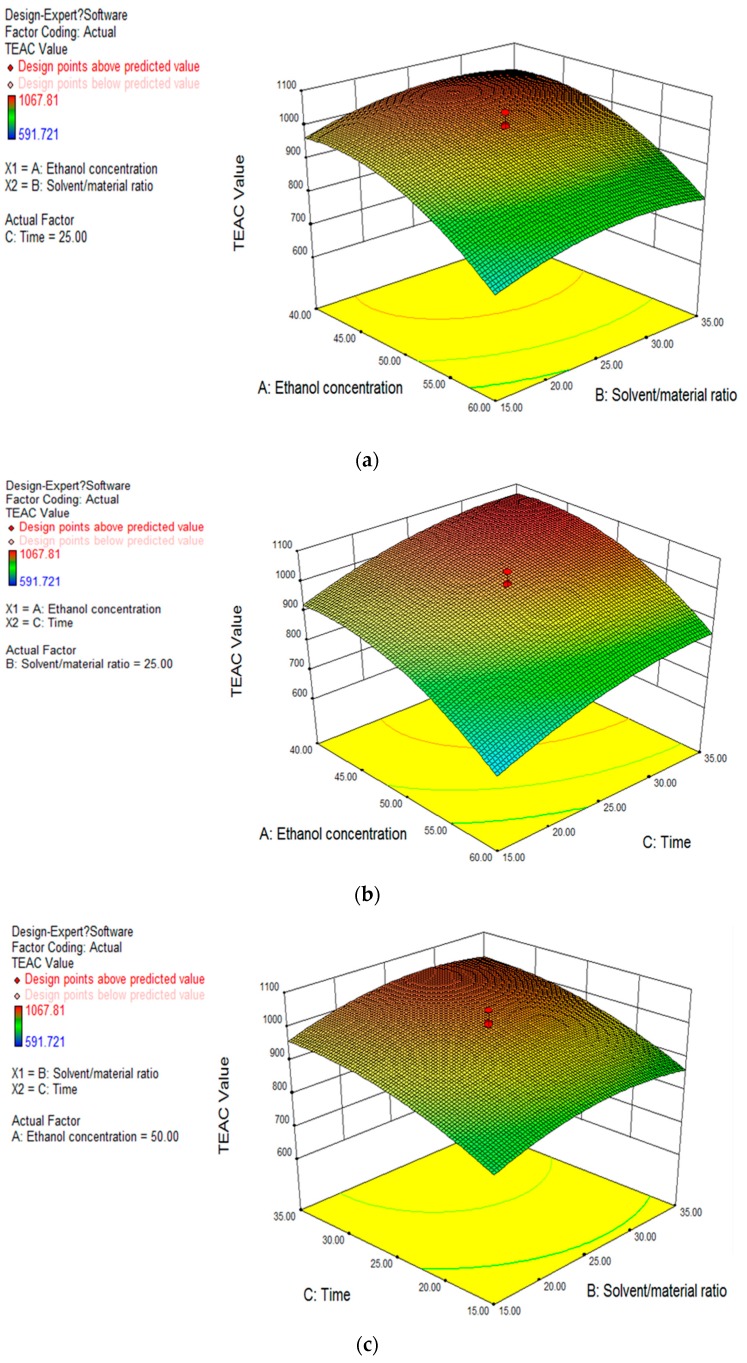
Response surface plots of the effects of solvent/material ratio (mL/g) and ethanol concentration (%) (**a**); extracting time (min) and ethanol concentration (**b**); and solvent/material ratio and extracting time (**c**) on TEAC value (μmol Trolox/g DW).

According to the results of Table 1, the three dimensional response surface plots were obtained, and are shown in Figure 2. The response surface plots were able to illustrate the relationship between the independent variables and the response value. As shown in Figure 2a, when the extracting time was fixed at 25 min, the response value (TEAC value) increased with decreasing ethanol concentration over a certain range, but when the ethanol concentration was reduced to nearly 40%, the response value was no longer increased. The response value increased with an increasing solvent/material ratio over a certain range, but when the solvent/material ratio was more than about 30 mL/g, the response value was no longer increased. In Figure 2b, when the solvent/material ratio was fixed at 25 mL/g, the response value increased with increasing extracting time over a certain range, but when the extracting time was increased to nearly 35 min, the response value was no longer increased. In Figure 2c, when the ethanol concentration was fixed at 50%, the effects of the solvent/material ratio and extracting time on the response value were similar to those of Figure 2a,b. According to the three response surface plots, the effects of ethanol concentration, solvent/material ratio, and extracting time on the response value, were not just simple linear effects; there were also quadratic effects. Therefore, each independent variable had an optimal value in the range. Under the condition of the optimal value, the response value had the maximum value. Based on the ANOVA and response surface plots, the ethanol concentration and extracting time had a more significant effect on the response value than the solvent/material ratio.

#### 2.2.4. Validation of Predicted Value

According to the response model, the optimal extraction conditions were obtained as follows: ethanol concentration of 42.98%, solvent/material ratio of 28.29 mL/g, extracting time of 34.29 min, temperature of 60 °C, and ultrasound power of 600 W. Under the optimal extraction conditions, the predicted value was 1077.37 μmol Trolox/g DW. In order to confirm the accuracy of the response model, a verification experiment with the optimal extraction conditions was performed, and the result showed that the TEAC value was 1074.61 ± 32.56 μmol Trolox/g DW. The result of the verification experiment was in agreement with the predicted value, which indicated that the response model was reliable and accurate. In addition, the TEAC value was 826.27 ± 9.07 μmol Trolox/g DW under the optimized extraction conditions without ultrasound, which indicated that ultrasound played an important role in improving the extraction efficiency.

### 2.3. Comparison of UAE with Conventional Extraction Methods

A comparative study was carried out between UAE and conventional extraction methods (maceration and Soxhlet extraction). The corresponding results are shown in Table 3. Compared with maceration extraction, UAE improved the extraction efficiency by 49%, and saved a lot of time. Compared with Soxhlet extraction, UAE improved the extraction efficiency by 1.07 times, and required a lower temperature and shorter time. The results were consistent with other reports on the extraction of natural antioxidants from black tea, walnut green husk, and the flower of *Limonium sinuatum* [39,40,41]. In addition, the total phenolic content (TPC) and total flavonoid content (TFC) of the extract obtained by UAE were significantly higher than those by maceration and Soxhlet extraction. The results indicated that UAE was the most effective method among the three extraction methods. The mechanical effect and cavitation induced by ultrasound disrupt the cell wall and increase mass transfer [42,43], which can explain the high efficiency of UAE.

### 2.4. Identification and Quantification of Phenolic Components

The identification of phenolic compounds helped to explain the strong antioxidant properties of the extract. So, the characterization of phenolic compounds in the extract obtained under optimal extraction conditions was performed by liquid chromatography-tandem mass spectrometry. Seven phenolic components were identified and quantified in the extract. As shown in Table 4, the highest phenolic component was epicatechin gallate, followed by epicatechin, rutin, epigallocatechin, protocatechuic acid, chlorogenic acid, and quercetin. Compared with other foods, the contents of some phenolic compounds in the *Melastoma sanguineum* Sims fruit were higher than those in other foods, and the contents of some phenolic compounds in the *Melastoma sanguineum* Sims fruit were lower than those in other foods [44,45,46]. For example, the contents of rutin and chlorogenic acid in the *Melastoma sanguineum* Sims fruit were 2.8 and 2.4 times higher, respectively, than those in whole grain quinoa [47], and the content of epicatechin in the *Melastoma sanguineum* Sims fruit was eight times higher than that in the *Thelephora ganbajun* mushroom [48]. However, the content of chlorogenic acid in Turkish coffee was 25 times higher than that in the *Melastoma sanguineum* Sims fruit [49], the content of epicatechin in *Aronia melanocarpa* Elliot was 1.7 times higher than that in the *Melastoma sanguineum* Sims fruit [50], the content of quercetin in mango was 7.8 times than that in the *Melastoma sanguineum* Sims fruit, and the contents of quercetin and chlorogenic acid in orange was 18.2 and 7.4 times higher, respectively, than that in the *Melastoma sanguineum* Sims fruit [44]. These phenolic components have a strong antioxidant activity [51,52,53], which might be part of the reason why the *Melastoma sanguineum* Sims fruit extract had a strong antioxidant capacity. In addition, these phenolic components have been proved to have various other biological activities, such as anti-inflammatory, antibacterial, anticancer, anti-hyperglycemia, hepatoprotective, and cardiovascular protective effects [54,55,56]. Therefore, the *Melastoma sanguineum* Sims fruit extract might have potential health benefits.

## 3. Materials and Methods

### 3.1. Chemicals and Reagents

2,2′-azinobis (3-ethylbenothiazoline-6-sulfonic acid) diammonium salt (ABTS), 6-hydroxy-2,5,7,8-tetramethylchromane-2-carboxylic acid (Trolox), Folin-Ciocalteu’s phenol reagent, gallic acid, and phenolic standards (chlorogenic acid, coffeic acid, daidzein, equol, epicatechin, epigallocatechin, epicatechin gallate, ferulic acid, genistein, glycitein, *p*-coumaric acid, protocatechuic acid, quercetin, rutin, and resveratrol) were purchased from Sigma-Aldrich (St. Louis, MO, USA). Potassium persulphate, aluminum chloride hexahydrate, potassium acetate, and sodium carbonate were purchased from Tianjin Chemical Factory (Tianjin, China). The formic acid and methanol used for chromatographic analysis were chromatographically pure. All other chemicals and reagents were analytically pure.

### 3.2. Sample Preparation

The mature fruits of *Melastoma sanguineum* Sims were manually harvested from many trees in the Lung Fu Mountain, Hong Kong, China, in October 2016. After picking, they were stored in a refrigerator at 4 °C, and used within one month. The fruits were cleaned using deionized water, and air dried in the room of a laboratory at room temperature, for about 4 h. The fruits were ground into fine particles by a special grinder with stainless steel knife (model JYL-C022E, Joyoung Co., Ltd., Jinan, Shandong, China), and stored in the dark at 4 °C.

### 3.3. Extraction of Natural Antioxidants

#### 3.3.1. Ultrasound-Assisted Extraction

The powder of *Melastoma sanguineum* Sims fruits (0.2 g) was put into a tube with an appropriate volume of ethanol aqueous solution. Then, the tube containing the mixture was placed into the water bath of an ultrasound device (Kj1012B; Kejin Ultrasonic Instrument Factory, Guangzhou, China), and was sonicated at a preset extraction time, temperature, and power. The ultrasonic energy introduced in the system was measured and calculated according to the literature [39,57], and was 12.5, 16.7, 19.6, 24.3, and 27.1 W/L, in response to the energy inputs to the generator of 400, 500, 600, 700, and 800 W, respectively. After sonication, the sample was centrifuged at 4200× *g* for 30 min, and the supernatant was collected for subsequent determination of antioxidant activity by spectrophotometry. In addition, the supernatant was filtered using a 0.45 μm membrane for the determination of phenolic compounds by liquid chromatography-tandem mass spectrometry.

#### 3.3.2. Maceration Extraction

The powder of the *Melastoma sanguineum* Sims fruits (0.2 g) was mixed with 5.658 mL of 42.98% ethanol, and extracted at 25 °C for 24 h in a shaking water bath. Then, the extraction solution was centrifuged at 4200× *g* for 30 min, and the supernatant was collected for subsequent determination.

#### 3.3.3. Soxhlet Extraction

The Soxhlet extraction process was carried out according to the method reported by Xu et al., with slight modifications [48]. The powder of the *Melastoma sanguineum* Sims fruits (1.0 g) was enveloped with Whatman filter paper, which was placed in a Soxhlet extractor. Then, 200 mL of 42.98% ethanol was used as the solvent, and heated at 95 °C. After 4 h of extraction in the Soxhlet extractor, the extraction solution was collected for subsequent determination.

### 3.4. Experimental Design

#### 3.4.1. Single Factor Experiment

The five factors (ethanol concentration, solvent/material ratio, extracting time, ultrasound temperature, and ultrasound power) were chosen to carry out a single factor experiment. The level gradients of each factor were: ethanol concentration 10%, 20%, 30%, 40%, 50%, 60%, 70%, 80%, 90%; solvent/material ratio 10, 15, 20, 25, 30, 35, 40, 45 mL/g; extracting time 10, 15, 20, 25, 30, 35, 40, 45 min; ultrasound temperature 30, 40, 50, 60, 70, 80 °C; ultrasound power 400, 500, 600, 700, 800 W.

#### 3.4.2. Response Surface Methodology

According to the results of the single factor experiment, three main factors (ethanol concentration, solvent/material ratio, and extracting time) were selected for RSM design. A three-factor, five-level CCRD, with 20 experimental runs was carried out. The independent variables and their five levels are presented in Table 5. The results of CCRD were analyzed using ANOVA, and fitted to a second-order polynomial equation, as follows:

Y = β_0_ + ∑β_i_X_i_ + ∑β_ii_X_i_^2^ + ∑β_ij_X_i_X_j_, (2)

In the equation, Y is the response value, X_i_ and X_j_ are the independent variables, β_0_ is the intercept, and β_i_, β_ij,_ and β_ii_ are the regression coefficients for the linear, interaction, and quadratic terms, respectively.

The multiple regression fitting of the experimental data and the ANOVA of the quadratic polynomial regression model were performed using Design Expert version 8.06.1 software (Stat-Ease, Minneapolis, MN, USA).

### 3.5. Determination of Antioxidant Capacity

Many methods have been developed to evaluate the antioxidant activity of foods and plants, and different methods could lead to a wide variation of antioxidant capacities [58]. However, the antioxidant capacities obtained using different methods usually have a very high correlationship [17,45]. Thus, the optimal extraction parameters obtained using different monitoring methods of antioxidant activity are usually the same or similar [59]. In the literature, a Trolox equivalent antioxidant capacity (TEAC) assay was usually selected for evaluating the antioxidant capacity of the extract, because it is a simple and rapid method, and can measure the antioxidant capacities of hydrophilic and lipophilic compounds in the same sample [17]. The TEAC assay was carried out based on the method previously established [60]. In brief, the ABTS^•+^ stock solution was prepared by mixing 2.45 mmol/L potassium persulfate solution and 7 mmol/L ABTS^•+^ solution with a ratio of 1:1 (*v*/*v*), and was then placed in the dark for 16 h and used within two days. The ABTS^•+^ working solution was obtained by diluting ABTS^•+^ stock solution, and the absorbance of ABTS^•+^ working solution was 0.70 ± 0.05 at λ_734 nm_. The 0.1 mL diluted sample was added to the 3.8 mL ABTS^•+^ working solution, and then incubated at room temperature for 6 min. After incubation, the absorbance of mixture was measured at λ_734 nm_, immediately. Trolox was employed as a reference standard, and the results were expressed as μmol Trolox/g DW of fruit.

### 3.6. Determination of Total Phenolic Content

The total phenolic content was determined on the basis of the method previously established [61]. In brief, a 0.50 mL diluted sample was added to 2.5 mL of 0.2 mol/L diluted Folin-Ciocalteu reagent. After 4 min of incubation, 2 mL saturated sodium carbonate solution was added. The absorbance of mixture was measured at λ_760 nm_ after 2 h of incubation at room temperature. Gallic acid was employed as a reference standard, and the results were expressed as mg of gallic acid equivalent (mg GAE)/g DW of fruit.

### 3.7. Determination of Total Flavonoid Content

The total flavonoid content was determined on the basis of the literature reported by Kalia et al. [62]. In brief, a 0.50 mL test sample was mixed with 1.5 mL of 95% ethanol (*v*/*v*), 0.1 mL AlCl_3_ solution (10%, *w*/*v*), 0.1 mL of 1 mol/L potassium acetate solution, and 2.8 mL double distilled water. The absorbance of mixture was determined at λ_415 nm_ after incubation at room temperature for 30 min. Quercetin was employed as the reference standard, and the results were expressed as mg quercetin equivalent (mg QE)/g DW of fruit. 

### 3.8. Identification and Quantification of Phenolic Compounds

Under optimal extraction conditions, the extract of the *Melastoma sanguineum* Sims fruit was obtained. The identification and quantification of phenolic compounds in the extract was performed by AB Sciex 4000 Qtrap liquid chromatography-tandem mass spectrometry (SCIEX, Framingham, MA, USA). The separation was performed by Acquity UPLC^®^ HSS T3 column (2.1 × 100 mm, 1.8 μm, Waters, Milford, MA, USA) at 40 °C. The mobile phase was composed of solution A (0.1% formic acid—water solution) and solution B (methanol), and the flow rate was 0.3 mL/min. The gradient elution was performed as follows: 15% B at 0–2 min, 15%–30% B at 2–8 min, 30%–80% B at 8–15 min, 80% B at 15–17.5 min, and 15% B at 17.5–19.5 min. The injection volume of the sample was 2 μL. The conditions of mass spectrometry were as follows: Ion source, ESI source with negative mode; Ion source temperature, 550 °C; detection mode, multiple reaction monitoring (MRM) mode; capillary voltage, −4500 V; curtain gas, 10 psig; nebulizer gas, 20 psig; auxiliary gas, 20 psig. Seven phenolic components were tentatively identified by tandem mass spectrometry. Then, they were verified by corresponding standard compounds, and quantified by peak areas.

### 3.9. Statistical Analysis

All of the experiments were carried out in triplicate, and the mean value ± standard deviation was reported. Statistical analysis was carried out using Design Expert 8.06.1 (Stat-Ease, Minneapolis, MN, USA) and SPSS 20.0 (IBM, Armonk, NY, USA). 

## 4. Conclusions

An UAE method has been developed for extracting natural antioxidants from the fruit of *Melastoma sanguineum* Sims, and RSM was employed for optimizing the extraction parameters. The high Adj. R^2^ (0.949) and consistency between the predicted value and experimental value indicated that the response model was reliable and accurate. The optimal extraction conditions were as follows: ethanol concentration of 42.98%, solvent/material ratio of 28.29 mL/g, extracting time of 34.29 min, temperature of 60 °C, and ultrasound power of 600 W. Under the optimal extraction conditions, the antioxidant capacity, TPC, and TFC of the extract were 1074.61 ± 32.56 μmol Trolox/g DW, 158.61 ± 6.44 mg GAE/g DW, and 2.58 ± 0.18 mg QE/g DW, respectively. Compared with conventional extraction methods, UAE was proved to be a more effective method for extracting natural antioxidants from the fruit of *Melastoma sanguineum* Sims. In addition, epicatechin gallate, epicatechin, rutin, epigallocatechin, protocatechuic acid, chlorogenic acid, and quercetin were identified and quantified in the extract, which might contribute to the strong antioxidant capacity of this fruit.

## Figures and Tables

**Table 1:** The experimental design, actual value, and predicted value, of response surface methodology.

Run	X_1_ (Ethanol Concentration, %)	X_2_ (Solvent/Material Ratio, mL/g)	X_3_ (Extracting Time, min)	Y (TEAC Value, μmol Trolox/g DW)	
				Actual Value	Predicted Value
1	50	25	25	988.49	998.34
2	50	25	25	981.79	998.34
3	40	35	15	876.92	905.11
4	60	35	35	855.01	846.94
5	40	35	35	1067.81	1050.23
6	66.82	25	25	591.72	604.16
7	50	25	25	1005.26	998.34
8	33.18	25	25	999.67	975.19
9	50	25	8.18	789.55	758.87
10	50	41.82	25	911.31	921.25
11	50	25	25	1045.49	998.34
12	60	15	35	799.64	779.95
13	50	25	41.82	997.43	1016.08
14	50	8.18	25	846.54	824.57
15	50	25	25	1008.61	998.34
16	60	35	15	689.15	676.78
17	60	15	15	593.10	619.19
18	50	25	25	958.32	998.34
19	40	15	35	971.98	992.86
20	40	15	15	840.55	857.13
validation	42.98	28.29	34.29	1074.61	1077.37

**Table 2:** Analysis of variance (ANOVA) of the response surface model.

Source	Sum of Squares	*df*	Mean Square	*F* Value	*p* Value	Significant
Model	368,043.36	9	40,893.71	40.59	<0.0001	significant
X_1_(Ethanol concentration)	166,172.90	1	166,172.90	164.93	<0.0001	
X_2_ (Solvent/material ratio)	11,282.75	1	11,282.75	11.20	0.0074	
X_3_ (Extracting time)	79,864.25	1	79,864.25	79.27	<0.0001	
X_1_X_2_	46.19	1	46.19	0.05	0.8348	
X_1_X_3_	313.39	1	313.39	0.31	0.5893	
X_2_X_3_	44.07	1	44.07	0.04	0.8385	
X_1_^2^	78,430.22	1	78,430.22	77.84	<0.0001	
X_2_^2^	28,340.63	1	28,340.63	28.13	0.0003	
X_3_^2^	22,140.13	1	22,140.13	21.97	0.0009	
Residual	10,075.50	10	1007.55			
Lack of Fit	5726.49	5	1145.30	1.32	0.3850	Not significant
Pure Error	4349.02	5	869.80			
Cor Total	378,118.86	19				
R-Squared	0.973					
Adj R-Squared	0.949					

**Table 3:** The comparison of UAE with conventional extraction methods.

Extracting Methods	Ethanol Concentration	Time	Temperature	TEAC Value (μmol Trolox/g DW)	TPC (mg GAE/g DW)	TFC (mg QE/g DW)
Maceration	42.98%	24 h	25 °C	723.27 ± 11.61	104.21 ± 2.17	1.39 ± 0.07
Soxhlet	42.98%	4 h	95 °C	518.37 ± 23.23	76.05 ± 2.71	1.89 ± 0.16
UAE	42.98%	34.29 min	60 °C	1074.61 ± 32.56	158.61 ± 6.44	2.58 ± 0.18

**Table 4:** The contents of phenolic components in extract.

Phenolic Components	Retention Time (*t*_R_, min)	Parent Ion (*m*/*z*, [M − H]¯)	Product Ion (*m*/*z*)	Contents (μg/g DW)
epicatechin gallate	6.87	441	169	1019.57 ± 99.39
epicatechin	5.4	289	203	90.34 ± 4.84
rutin	9.67	609	300	61.6 ± 1.59
epigallocatechin	3.03	305	137	28.67 ± 2.62
protocatechuic acid	3.09	152.9	107.8	3.22 ± 0.23
chlorogenic acid	4.13	353	191	2.34 ± 0.28
quercetin	11.8	301	179	1.39 ± 0.18

**Table 5:** Independent variables and their five levels used for central composite rotatable design.

Independent Variable	Units	Symbol	Coded Levels				
			−1.68	−1	0	1	1.68
Ethanol concentration	% (*v*/*v*)	X_1_	33.18	40	50	60	66.82
Solvent/material ratio	mL/g	X_2_	8.18	15	25	35	41.82
Extracting time	min	X_3_	8.18	15	25	35	41.82
